# Armour® Thyroid Rage - A Dangerous Mixture

**DOI:** 10.7759/cureus.2523

**Published:** 2018-04-24

**Authors:** Joshua Coutinho, Justin B Field, Anupam A Sule

**Affiliations:** 1 Internal Medicine, St. Joseph Mercy Oakland Hospital, Oakland, USA

**Keywords:** drug interactions, levothyroxine, testosterone replacement therapy, stemi, myocardial infarction, hypercoagulability, armour thyroid, thrombosis, st-segment elevation myocardial infarction

## Abstract

Armour® Thyroid (Forest Pharmaceuticals, LLC; affiliate of Allergan, Dublin, Ireland) is a natural porcine derivative thyroid supplement that is frequently used without physician monitoring by health enthusiasts as a weight loss supplement. Although there are no publications associating Armour Thyroid and major coronary events, significant drug interactions may exist.

A 32-year-old male with a history of hypothyroidism, cystic acne, and solitary congenital kidney presented to the emergency room after experiencing crushing substernal chest pain radiating to his left shoulder, accompanied by diaphoresis and shortness of breath. The patient denied any tobacco use or family history of heart disease. He was self-administering 120 mg of Armour Thyroid daily.

On examination, the patient was well-developed with cystic acne and a flushed appearance. His vital signs on admission were a blood pressure of 171/106 mmHg, heart rate of 88 beats per minute (bpm), and respiratory rate of 16 breaths/min. The electrocardiogram revealed marked ST-segment elevation in the anterior chest leads. Laboratory studies revealed elevated troponins. Urine drug screen was negative. The patient underwent an emergent coronary angiogram, which confirmed an occluded left anterior descending artery. He was treated successfully by thrombectomy and stenting of his left anterior descending artery.

Evaluation for other causes of thrombosis was negative: glycosylated hemoglobin (HbA1C) 5.5%, low-density lipoprotein (LDL) 127 mg/dL, high-density lipoprotein (HDL) 33 mg/dL, hypercoagulable evaluation negative, and hemoglobin (Hgb) 17.1 gm/dL. Luteinizing hormone (LH) and follicle stimulating hormone (FSH) were < 0.20 miu/mL. Thyroid profile results were thyroid-stimulating hormone (TSH) 0.20 miu/mL (low), T3 free 4.08 pg/mL (high), and T4 total 1.2 mcg/dL (low), which were consistent with exogenous thyroid hormone administration.

Focused questioning triggered by his cystic acne led to the discovery that the patient was self-administering exogenous testosterone replacement therapy. The patient declined to share specifics with the healthcare team. This was confirmed by a high testosterone level of 1,311 ng/dL.

Hyperthyroidism increases the risk of cardiovascular events two to three times through the propagation of a hypercoagulable, hypofibrinolytic state possibly via an increase in clotting factors, a decrease in fibrinolytic enzymes, and an increased inhibition of the protein C pathway. The effect of androgens on cardiovascular mortality is uncertain. Androgens stimulate the hemostatic system, increase adverse lipid profile, and erythropoiesis. The combined therapy likely resulted in a synergistic potentiation of hypercoagulable, hypofibrinolytic effects of both agents. Given the absence of other cardiovascular risk factors, the cause of the myocardial infarction in our patient was likely due to drug interaction between Armour Thyroid and exogenous testosterone therapy.

Due to the potential drug interaction between both natural and prescribed thyroid hormone and testosterone supplements, patients should be discouraged from self-administration of thyroid or anabolic steroids. Due to the lack of standardization in the T3 content, the use of Armour Thyroid should be avoided.

## Introduction

An increased incidence of myocardial infarction has been reported in patients suffering from hyperthyroidism due to the pro-coagulant effects of thyroid hormone [1]. On the other hand, levothyroxine supplementation based upon physiological requirements under medical supervision has been demonstrated to be safe with no increase in cardiac events [2]. Armour® Thyroid (Forest Pharmaceuticals, LLC; affiliate of Allergan, Dublin, Ireland) is a crushed porcine thyroid derivative often used by health enthusiasts as a weight loss supplement. There are no reports of Armour Thyroid causing cardiovascular events.

There is conflicting evidence about the effect of testosterone hormone supplementation on myocardial infarction with some studies suggesting a cardioprotective effect of testosterone supplementation [3]. On the other hand, case reports have been published that describe sudden death in athletes using androgenic steroids [4]. 

## Case presentation

A 32-year-old male presented to the emergency department with 20 mins of cramping retrosternal chest pain radiating to his left shoulder accompanied by sweating and shortness of breath. He did not have a history of any cardiovascular risk factors, such as a history of smoking, diabetes, or hypertension. He did not have any family history of cardiac events in family members at an early age. He had a self-reported diagnosis of hypothyroidism for which he was self-administering 120 mg of Armour Thyroid daily.

**Figure 1:**
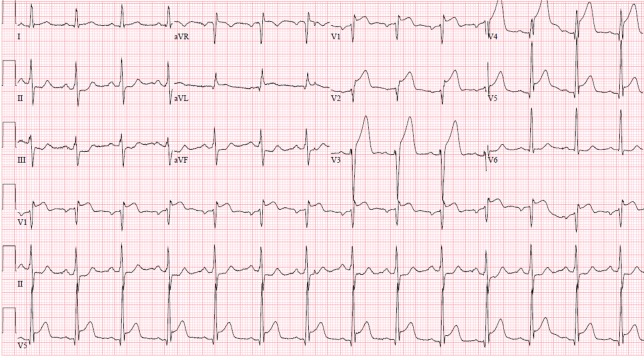
Electrocardiogram at presentation demonstrating ST elevation myocardial infarction in the anterior leads

At the time of presentation, his blood pressure was 171/106 mm of Hg, heart rate was 88 beats per minute, and respiratory rate was 16 breaths per minute. Physical exam was notable for well-developed musculature and cystic acne. Other physical examination findings were unremarkable. A 12-lead electrocardiogram (ECG) (Figure 1) demonstrated ST-segment elevations in leads aVL, I, and v1-v6, as well as ST segment depressions in leads II, III, and aVF, suggestive of an acute ST elevation myocardial infarction (STEMI). 

Initial lab work reported markedly increased levels of cardiac troponin. Urine drug screen was negative, eliminating cocaine as a potential etiology.

**Figure 2:**
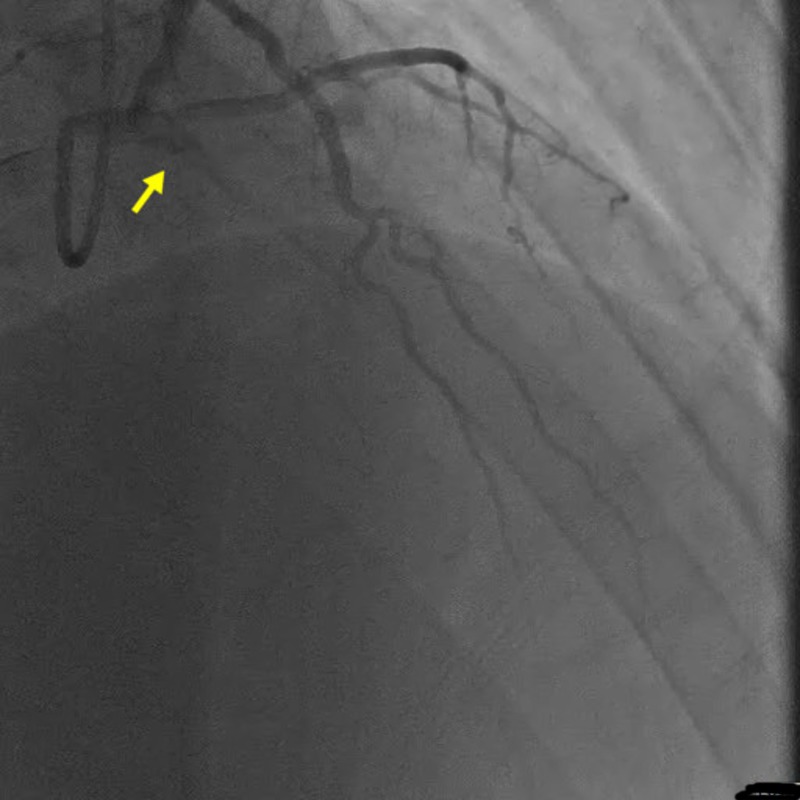
Coronary angiogram revealing complete occlusion of LAD coronary artery at the ostium LAD: left anterior descending

Transthoracic echocardiography (TTE) displayed a moderate increase in left ventricular (LV) wall thickness, reduced ejection fraction (EF) of 40%, grade 1 diastolic dysfunction, and hypokinetic anterior and anteroseptal walls in the distribution of the left anterior descending (LAD) coronary artery. Emergent left heart catheterization was performed via the right radial artery using the Seldinger technique. An LV pressure of 117/5 mm of Hg with an LV end-diastolic pressure of 14 mm of Hg was noted. A coronary angiogram revealed a complete occlusion of the LAD at the ostium (Figure 2).

**Figure 3:**
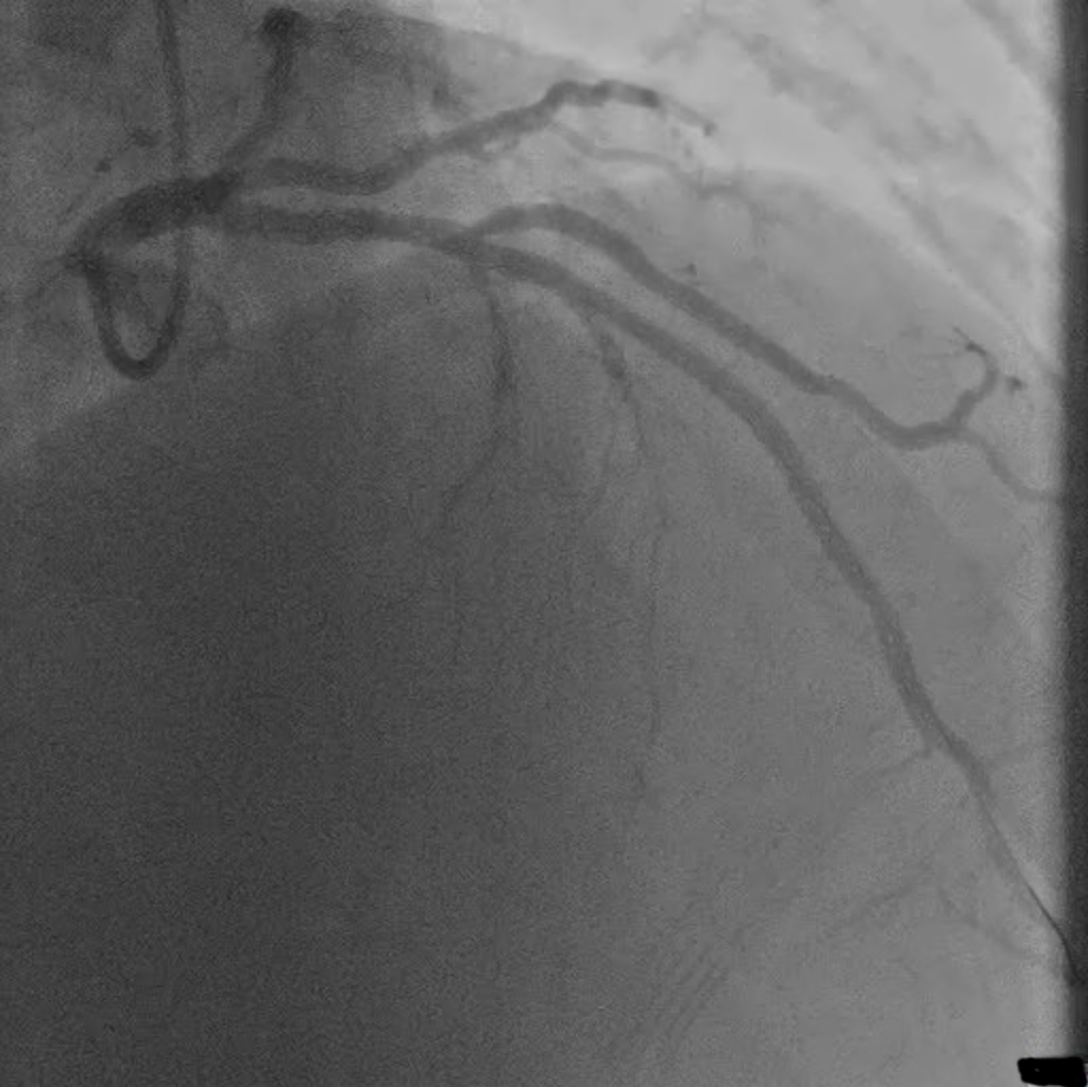
Coronary angiogram confirming coronary flow after intervention

The remainder of the coronary arteries were patent without evidence of atherosclerotic changes. Manual thrombectomy of the LAD was performed, and a XIENCE Alpine 3.25 mm x 15 mm drug-eluting stent (Abbott Laboratories; Abbott Park, IL, USA) was positioned leading to return of TIMI-III flow (Figure 3).

The patient was started on dual antiplatelet therapy with aspirin and clopidogrel, in addition to heparin and eptifibatide infusions. His subsequent fasting lipid profile was normal with low-density lipoprotein of 127 mg/dL, a high-density lipoprotein of 31 mg/dL, and triglycerides of 44 mg/dL. Focused questioning to elicit the potential cause of the myocardial infarction led to the revelation that the patient participated in recreational bodybuilding for which he self-administered exogenous testosterone therapy and was using Armour Thyroid as a weight loss supplement.

His testosterone levels were elevated at 1,311 ng/dL with luteinizing hormone (LH) and follicle stimulating hormone (FSH) levels below trace levels of 0.20 mIU/mL, confirming exogenous testosterone supplementation. Free triiodothyronine (T3) was high at 4.08 pg/mL with a suppressed total thyroxine (T4) at 1.2 mcg/dL and a thyroid stimulating hormone (TSH) at 0.20 mIU/mL, confirming Armour Thyroid administration.

## Discussion

Prior studies of testosterone supplementation have reported conflicting data [3-4]. Some studies have reported worsening of the lipid profile, while others reported improvement. Similarly, some studies have reported improvement in angina symptoms, while others did not report any effect. Testosterone supplementation may lead to increased activation of the hemostatic system with elevations in prothrombin, antithrombin III, and protein S, and a decrease in the tissue plasminogen activator and its inhibitor. Exogenous testosterone replacement therapy has been associated with the development of venous emboli through directly induced polycythemia, hypertension, and hyperlipidemia but a causation for cardiovascular events has not been established [5].

Hyperthyroidism has been associated with a higher incidence of coronary artery disease and acute cardiovascular events [6]. Increased levels of free thyroxine hormone lead to increased concentrations of clotting factors and fibrinogen with a decrease in tissue plasminogen activator leading to a hypercoagulable, hypofibrinolytic environment [1]. Levothyroxine supplementation in hypothyroidism has not been associated with an increased risk of acute cardiovascular events [2].

Testosterone affects thyroid hormone carrier proteins causing a decrease in thyroxine-binding globulin, thyroid stimulating hormone, and total T4 but an increase in free T4 and T3 [7]. The patient demonstrated a similar pattern for his thyroid hormone tests. Armour Thyroid is a porcine desiccated thyroid-derived supplement that was being used by the patient for weight loss. Due to the lack of standardization, there might have been significant batch-to-batch variation in the number of active ingredients being consumed by the patient. The co-self-administration of Armour Thyroid and testosterone in this patient possibly led to the development of a hypercoagulable, hypofibrinolytic milieu with alterations in the clotting factors and polycythemia. This would have accelerated the development of a thrombus in his LAD in the absence of underlying atherosclerosis in his coronary arteries.

## Conclusions

Coadministration of Armour Thyroid and testosterone may cause thromboembolic events.

Physicians prescribing testosterone and thyroid hormone supplementation should consider the potential for drug interaction of these medications and adjust the levothyroxine dose based on frequent serum thyroid hormone level monitoring.
